# Breastfeeding woman are at higher risk of vitamin D deficiency than non-breastfeeding women - insights from the German VitaMinFemin study

**DOI:** 10.1186/s13006-017-0105-1

**Published:** 2017-04-19

**Authors:** Sandra Gellert, Alexander Ströhle, Andreas Hahn

**Affiliations:** Leibniz University Hannover, Am Kleinen Felde 30, 30167 Hannover, Germany

**Keywords:** Vitamin D, 25(OH)D, Breastfeeding period, Germany

## Abstract

**Background:**

Despite increased awareness of the adverse health effects of vitamin D deficiency, only a few studies have evaluated the vitamin D status (25-hydroxyvitamin D [25(OHD)]) of breastfeeding women and up to now, no information exits for German breastfeeding women. Therefore, the aim of study was to determine the vitamin D status of breastfeeding women compared to non-pregnant and non-breastfeeding (NPNB) women.

**Methods:**

This cross-sectional study investigated 124 breastfeeding women and 124 age and season matched NPNB women from the German “Vitamin and mineral status among German women” study. The study participants were recruited from April 2013 to March 2015 and did not take vitamin D supplements. Serum 25(OH)D was analyzed by chemiluminescent immunoassay.

**Results:**

Vitamin D deficiency (<25.0 nmol/L) was prevalent in 26.6% of the breastfeeding women. The majority of women (49.2%) showed 25(OH)D concentration between 25.0 and 49.9 nmol/L. In multiple binary logistic regression analysis, breastfeeding women had a 4.0-fold higher odds ratio (OR) (95% confidence interval [CI] 1.8, 8.7) for vitamin D deficiency than NPNB women. For breastfeeding women, the risk of vitamin D deficiency was higher in the winter and spring months (OR: 2.6, 95% CI 1.1, 6.3) and increased with lower longitude per one unit (OR 0.7, 95% CI 0.6, 0.9).

**Conclusion:**

Breastfeeding women in Germany had a higher risk of deficient vitamin D levels than NPNB women. In further studies, the optimal vitamin D status for breastfeeding women should be investigated and also the required vitamin D doses to ensure this vitamin D status.

**Trial registration:**

German Clinical Trial Register (identification number: DRKS00004789).

## Background

Vitamin D deficiency is common in Europe [1]. In Germany, an estimated 56.1% of non-breastfeeding women have insufficient vitamin D status (<50 nmol/L) with a higher prevalence in winter [2]. The vitamin D requirement can be ensured by endogenous synthesis in the skin via ultraviolet B (UVB) radiation and therefore depends on numerous factors that affect the synthesis rate [3]. Vitamin D is widely recognized as a factor that not only affects calcium and bone metabolism [4] but may also be protect against some diseases such as musculoskeletal health problems, various autoimmune disorders, cardiovascular disease and cancer [5].

Breastfeeding represents a critical period in regards to vitamin D status in the lifecycle of a woman. A total of 49.0% of German pregnant women in the summer and 98.0% of pregnant women in the winter show inadequate vitamin D status after the birth of a child [6], indicating that the breastfeeding period often starts with a maternal vitamin D deficit. Moreover, the maternal vitamin D status affects not only her own health but also that of her breastfed infant [7]. Data suggest that the 25-hydroxyvitamin D (25(OH)D) content of breast milk correlates with maternal 25(OH)D status [8]. Therefore, the transfer of vitamin D through breast milk might have a negative impact on the maternal vitamin D status. In addition, there will also be negative consequences for the infant, as the transfer of vitamin D through milk may not be sufficient to satisfy infant nutritional requirements if the breastfeeding woman is vitamin D deficient [7]. Inadequate vitamin D status should be avoided to prevent rickets [9] and other diseases such as respiratory infections [10] and heart failure [11] in infants. In addition, rickets increases the risk of type 1 diabetes [12]. Nevertheless, in Germany, the recommended vitamin D intake of 800 international units (IU)/day during the breastfeeding period is the same as that for non-breastfeeding women [13]. Moreover, the content of the mother’s milk is insufficient to ensure the vitamin D requirement of the infant [8]. Irrespective of whether the infant is breastfed, the estimated value of vitamin D intake for infants up to one year of age is 400 IU/day in Germany [13]. Recent findings show that maternal vitamin D supplementation of 6400 IU/day during the breastfeeding period, rather than vitamin D supplementation for the infant, is effective to ensure the requirement of the infant [14].

Vitamin D levels in healthy adults have been reported in several studies [1, 2, 15]. However, only a few studies have evaluated vitamin D status in breastfeeding women [16–22]. Moreover, until now, information regarding vitamin D status in breastfeeding women has not been available in Germany.

This study addresses this gap in knowledge by examination of vitamin D status in breastfeeding women in comparison with a group of age and season matched women who were not pregnant and did not breastfeed (NPNB) in a nationwide, cross-sectional multicenter study. In addition, factors associated with vitamin D status among breastfeeding women were examined.

## Methods

### Study design and participants

This study sample was obtained from the nationwide, cross-sectional, multicenter VitaMinFemin study (Vitamin and mineral status among German women), which analyzed the status of selected nutrients in women at different stages of life (*n* = 2367).

Study participants were recruited from April 2013 (first participant in) to March 2015 (last participant in) in cooperation with 125 study sites (latitude 47.6°N to 54.2°N, longitude 6.3°E to 13.9°E). Details of the study design and implementation have been previously described [23].

**Fig. 1:**
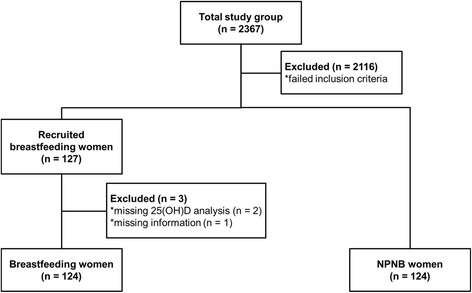
Recruitment of study participants. 25(OH)D, 25-hydroxyvitamin D; NPNB, non-pregnant and non-breastfeeding

Of the 2367 women recruited at different stages of life, 124 women met the inclusion criteria (breastfeeding women) and did not fulfill any exclusion criteria (Fig. 1). The cross-sectional analysis in breastfeeding women was performed at different time points postpartum (between two weeks and nine months postpartum) and corresponded to the respective time since the beginning of the breastfeeding period in all participants. A comparison group of NPNB women (*n* = 124) was also selected from the VitaMinFemin study and matched by age and season of blood sampling. No one in either group supplemented with vitamin D.

### Data collection

Data collection included questionnaire and blood sample which were conducted on the same day. Using a questionnaire, age, anthropometric variables (height and weight), skin type (light/dark), holidays within the six weeks before data collection (yes/no) in countries south of Germany where the sunshine is sufficient to produce endogenous vitamin D, smoking status (yes/no) and time since the beginning of breastfeeding period were included. Serum 25(OH)D levels were measured at the LADR laboratory, Germany.

### 25(OH)D measurement

Vitamin D status was measured once per participant in serum 25(OH)D, which is considered to be the best indicator and reflects both vitamin D intake and endogenous vitamin D synthesis [24]. Chemiluminescence immunoassay (LIASION® TOTAL Assay, DiaSorin Inc., Stillwater, MN, USA) was used for 25(OH)D analysis. The lower and higher detection limits of the analysis were 10 and 375 nmol/L without dilution, respectively. Quality was ensured in accordance with DIN ISO 15189.

### Cutoff values for 25(OH)D

Subgroups were categorized based on their 25(OH)D concentration. In the general population, as well as in the breastfeeding woman, no current recommendations exist regarding optimal serum 25(OH)D levels [5, 7, 24, 25]. Therefore, we used a common classification scheme to determine vitamin D status, as utilized by Richter et al. [15] and described as follows: (1) severe deficiency (<15.0 nmol/L) as used previously [26]; (2) moderate deficiency (15.0-24.9 nmol/L, corresponding to a deficit threshold of <25.0 nmol/L) [27], which is associated with osteomalacia, severe hyperparathyroidism [27], myopathy and rickets in breastfeeding infants [28]; (3) insufficiency (25.0–49.9 nmol/L) [27], which leads to impaired muscle function, reduced bone mineral density and elevated parathyroid hormone (PTH) levels [27]; (4) sufficiency (50.0–74.9 nmol/L) [27] with low vitamin D stores and slightly increased PTH levels [27] and (5) optimal vitamin D status (75.0–124.9 nmol/L, corresponding to an optimal threshold of >75.0 nmol/L) [29] where vitamin D dependent functions are not impaired [27] and the mortality risk in colorectal or breast cancer is reduced [30]. Additionally, we considered (6) ≥125.0 nmol/L to be the threshold at which there is a risk of vitamin D excess [24], as the morality risk is increased above this level [31].

### Influencing factors of serum 25(OH) D values

The 25(OH)D value of breastfeeding women was assessed relative to that of NPNB women. As several factors have been found to be related to 25(OH)D levels [2, 15, 32], the 25(OH)D status was also evaluated with the inclusion of the following potential confounding variables: (a) “season at the time of blood collection” with the classification of participants in spring (March to May), summer (June to August), autumn (September to November) and winter (December to February); (b) “region” (i.e., latitude and longitude where people were recruited); (c) recent “holidays” in the last six weeks before blood sampling; (d) “skin type” (light/dark) using a modified schema of the pigmentation classification by Fitzpatrick [33]; (e) “age”; (f) “body mass index” (BMI); (g) “smoking” and (h) “duration of breastfeeding” to analyze whether the duration of breastfeeding had an effect on the vitamin D status.

### Statistical analysis

Statistical package for social science (SPSS) software version 22.0 (SPSS, Inc. Chicago, Illinois, USA) was used for statistical analyses. Continuous variables are presented as mean ± standard deviation (SD) and range (minimum, maximum) and categorical variables as number of participants (*n*) and percentage (%). Significant differences between breastfeeding women and NPNB women were analyzed using nonparametric Mann-Whitney *U*-test due to the skewed distribution of the data and differences between categorical variables were analyzed using Chi-square test. Spearman’s rank correlation was used to test correlations between variables with skewed distributions and *p* values ≤0.05 were considered significant. Univariate and multivariate binary logistic regressions were performed to assess potential associations between variables and the odds ratio (OR) of vitamin D deficiency. A threshold 25.0 nmol/L was used to define vitamin D deficiency (<25.0 nmol/L) and non-deficiency (≥25.0 nmol/L). The potential determinants season, skin type, recent holiday and smoking were included in the model as categorical variables. Reference categories were defined as those with the lowest assumed prevalence rate of vitamin D deficiency. The ORs for region (latitude and longitude), age, BMI and duration of breastfeeding were evaluated to determine the increase in the odds of vitamin D deficiency per increase in variable unit. The multivariate binary logistic regression included only determinants with *p* values <0.05 in the univariate binary logistic regressions.

## Results

### Baseline characteristics

Table 1 presents the characteristics of the study sample for breastfeeding women and NPNB women separately. Age, body height, body weight, BMI and prevalence of season of blood sampling, skin type and recent holidays did not differ between breastfeeding women and NPNB women. The prevalence of smoking was higher in NPNB women (24.2%) than in breastfeeding women (8.9%, *p* = 0.001). A total of 24.6% of the study sample were overweight (BMI 25–29.9 kg/m^2^) and 12.5% were obese (BMI ≥30.0 kg/m^2^). The percentage of overweight and obese did not differ between breastfeeding women and NPNB women (*p* = 0.093), data not shown.

**Table 1:** Characteristics of study sample

		Breastfeeding women (*n* = 124)	NPNB women (*n* = 124)	*P* value
Age (years)	Mean ± SD	31.9 ± 5.0	31.8 ± 5.3	0.784^‡^
	Range	20.0 − 44.0	19.0 − 45.0	
Height (m)	Mean ± SD	1.68 ± 0.07	1.67 ± 0.07	0.663^‡^
	Range	1.52 − 1.86	1.52 − 1.82	
Weight (kg)	Mean ± SD	71.2 ± 14.5	68.4 ± 14.9	0.064^‡^
	Range	48.0 − 142.0	43.0 − 120.0	
BMI (kg/m^2^)	Mean ± SD	25.1 ± 4.8	24.3 ± 5.0	0.053^‡^
	Range	18.2 − 46.4	17.0 − 40.8	
Season of blood sampling				
Spring	*N* (%)	22 (17.7)	22 (17.7)	1.000^*^
Summer		14 (11.3)	14 (11.3)	
Autumn		63 (50.8)	63 (50.8)	
Winter		25 (20.2)	25 (20.2)	
Skin type				
Light	*N* (%)	109 (87.9)	112 (90.3)	0.292*
Dark		15 (12.1)	10 (8.1)	
Recent holidays	*N* (%)	5 (4.0)	6 (4.8)	0.758*
Smoking	*N* (%)	11 (8.9)	30 (24.2)	0.001*
Duration of breastfeeding (month)^a^	Mean ± SD	3.5 ± 3.2	/	/
	Range	0.5 − 18.0		

Autumn, September – November; BMI, body mass index; NPNB, non-pregnant and non-breastfeeding; SD, standard deviation; Spring, March – May; Summer, June – August; Winter, December – February

^‡^Mann-Whitney *U*-test

*Chi-square test

^a^ *n* = 123

### Vitamin D status

**Fig. 2:**
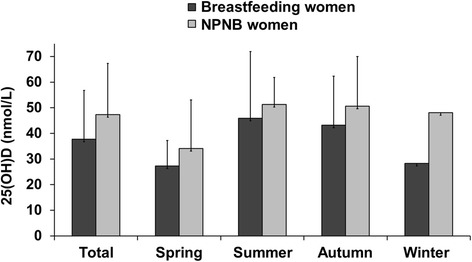
25-hydroxyvitamin D concentration in breastfeeding women compared to NPNB women. Bars indicate mean ± standard deviation; Breastfeeding vs. non-breastfeeding women in total study sample: *p* <0.001^‡^; Between season in breastfeeding women: *p* < 0.001^†^; Between season in NPNB women: *p* = 0.002^†^; Breastfeeding vs. NPNB women in spring: *p* = 0.391^‡^, summer: *p* = 0.352^‡^, autumn: *p* = 0.026^‡^, winter: *p* = 0.001^‡^; Autumn, September – November; NPNB, non-pregnant and non-breastfeeding; Spring, March – May; Summer, June – August; Winter, December – February; ^‡^Mann-Whitney *U*-test, †Kruskal-Wallis-test

In breastfeeding women, the mean 25(OH)D concentration (37.7 ± 19.1 nmol/L) was significantly lower than in NPNB women (47.3 ± 20.0 nmol/L, *p* <0.001) (Fig. 2). Serum 25(OH)D varied significantly by season for both breastfeeding and NPNB women. Breastfeeding women had the highest 25(OH)D levels in summer (45.9 ± 26.0 nmol/L) and autumn (43.2 ± 19.1 nmol/L) and the lowest 25(OH)D levels in winter (28.3 ± 13.0 nmol/L) and spring (27.3 ± 9.9 nmol/L; *p* <0.001).

In autumn and winter, levels of 25(OH)D were significantly lower in breastfeeding women (43.2 ± 19.1 nmol/L; 28.3 ± 13.0 nmol/L) than in NPNB women (50.6 ± 19.4 nmol/L, *p* = 0.026; 48.1 ± 22.4 nmol/L, *p* = 0.001). In the spring and summer, there were no differences in 25(OH)D concentration between breastfeeding women and NPNB women (spring: *p* = 0.391, summer: *p* = 0.352).

The 25(OH)D level was not significantly associated with any of the possible factors in breastfeeding women (latitude of residence, skin type, age, BMI, month of breastfeeding and smoking), data not shown. However, 25(OH)D concentrations in breastfeeding women had a weak positive association with longitude of residence (*r*_*s*_ = 0.263, *p* = 0.003).

### Prevalence of 25(OH)D categories between breastfeeding and NPNB women

**Fig. 3:**
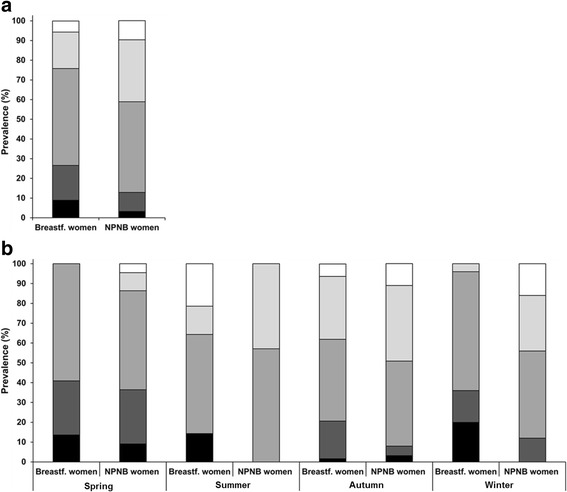
Prevalence of vitamin D status by (**a**) stage of life and (**b**) season. Classification of vitamin D status (25(OH)D concentration) according to  severe deficiency (<15.0 nmol/L),  moderate deficiency (15.0–24.9 nmol/L),  insufficiency (25.0–49.9 nmol/L),  sufficiency (50.0–74.9 nmol/L) and optimal (75.0–124.9 nmol/L); Risk of excess (≥124.9 nmol/L) was not prevalent; (**a**) Breastfeeding women vs. NPNB women: *p* = 0.019*; (**b**) Between season in breastfeeding women: *p* <0.001*; Between season in NPNB women: *p* = 0.032*; Breastfeeding women vs. NPNB women in spring: *p* = 0.498*, summer: *p* = 0.070*, autumn: *p* = 0.139*, winter: *p* = 0.007*; 25(OH)D, 25-hydroxyvitamin D; Autumn, September – November; NPNB, non-pregnant and non-breastfeeding; Spring, March – May; Summer, June – August; Winter, December – February; *Chi-square test

Frequencies of 25(OH)D levels in the previously defined target ranges are shown in Fig. 3. The prevalence of vitamin D deficiency (<25.0 nmol/L) and insufficiency (<50.0 nmol/L) was significantly higher in breastfeeding women (26.6% and 75.8%, respectively) than in NPNB women (12.9%, *p* = 0.007 and 58.9%, *p* = 0.004, respectively). In contrast, only 5.6% of the breastfeeding women and 9.7% of the NPNB women had optimal 25(OH)D levels (75.0–124.9 nmol/L). No participant had a risk of vitamin D excess (≥125.0 nmol/L).

The frequencies of the 25(OH)D categories depended on the season of blood sampling in both groups. In breastfeeding women, the proportion having optimal vitamin D levels (75.0–124.9 nmol/L) was the highest in summer, whereas in winter, none of the breastfeeding women had optimal 25(OH)D concentrations. In spring, every women had 25(OH)D levels below the sufficient value (<50.0 nmol/L).

Breastfeeding women had a significantly higher prevalence of vitamin D deficiency (<25.0 nmol/L) in autumn and winter and a lower prevalence of optimal vitamin D levels (75.0–124.9 nmol/L) in winter than NPNB women.

### Risk factors for vitamin D deficiency

In breastfeeding women, the risk for vitamin D deficiency was significantly positively associated with the winter and spring months, lower longitude of place of residence and higher BMI in the univariate binary logistic regression (Table 2). In contrast, no significant association was found between latitude, skin type, recent holidays, smoking, age, duration of breastfeeding and vitamin D deficiency.

**Table 2:** Univariate odds ratios for vitamin D deficiency (<25.0 nmol/L) in breastfeeding women

Determinants	*N*	Persons at risk (% of category)	*P* value	Odds ratios	95% CI	*P* value
Season						
Summer and Autumn	77	15 (19.5)	0.021*	1.0	Ref.	
Winter and Spring	47	18 (38.3)		2.6	1.1, 5.8	0.023
Region^a^						
Latitude	/	/	/	1.1	0.9, 1.3	0.378
Longitude	/	/	/	0.7	0.6, 0.9	0.006
Skin type						
Light	109	29 (26.6)	0.996*	1.0	Ref.	
Dark	15	4 (26.7)		1.0	0.3, 3.4	0.996
Recent holidays						
Yes	5	1 (20.0)	0.733*	1.0	Ref.	
No	119	32 (26.9)		0.7	0.2, 13.7	0.734
Smoking						
No	113	30 (26.5)	0.959*	1.0	Ref.	
Yes	11	3 (27.3)		1.0	0.3, 4.2	0.959
Age^a^	/	/	/	0.9	0.9, 1.0	0.163
BMI^a^	/	/	/	1.1	1.0, 1.2	0.045
Duration of breastfeeding (month)^b^	/	/	/	1.0	1.0, 1.0	0.703
Stage of life						
Breastfeeding women	124	16 (12.9)	0.007*	1.0	Ref.	
NPNB women	124	33 (26.6)		2.5	1.3, 4.7	0.008

25(OH)D, 25-hydroxyvitamin D; Autumn, September – November; BMI, body mass index; CI, confidence interval; NPNB, non-pregnant and non- breastfeeding; Ref., reference category with the lowest assumed prevalence of vitamin D deficiency; Spring, March – May; Summer, June – August; Winter, December – February

*Chi-square test for prevalence differences of 25(OH)D concentrations below 25 nmol/L

^a^Odds ratio for an increase per one unit

^b^ *n* = 123

The determinants of vitamin D deficiency (<25.0 nmol/L) by multiple binary logistic regression analysis are shown in Table 3. Breastfeeding woman had a 4.0-fold greater odds ratio of vitamin D deficiency than NPNB women (*p* = 0.001). In breastfeeding women, the odds increased significantly in the winter and spring months (OR = 2.6; *p* = 0.029) compared to the summer and autumn months. Longitude was an important determinant of serum 25(OH)D; the OR for vitamin D deficiency lies at 0.7 for each increase in longitude unit (*p* = 0.004).

**Table 3:** Multivariable adjusted odds ratios for vitamin D deficiency (<25.0 nmol/L) in breastfeeding women

Determinants	*N*	Persons at risk (% of category)	*P* value	Odds ratios	95% CI	*P* value
Season^a^						
Summer and Autumn	77	15 (19.5)	0.021*	1.0	Ref.	
Winter and Spring	47	18 (38.3)		2.6	1.1, 6.3	0.029
Region^b,c^						
Longitude	/	/	/	0.7	0.6, 0.9	0.004
BMI^c,d^	/	/	/	1.1	1.0, 1.2	0.060
Stage of life^e^						
Breastfeeding women	124	16 (12.9)	0.007*	1.0	Ref.	
NPNB women	124	33 (26.6)		4.0	1.8, 8.7	0.001

25(OH)D, 25-hydroxyvitamin D; Autumn, September - November; BMI, body mass index; CI, confidence interval; NPNB, non-pregnant, non-breastfeeding; Ref., reference category with the lowest assumed prevalence of vitamin D deficiency; Spring, March – May; Summer, June – August; Winter, December – February

*Chi-square test for prevalence differences of 25(OH)D concentrations below 25 nmol/L

^a^Multiple binary regressions considering the terms region (longitude) and BMI

^b^Multiple binary regressions considering the terms season and BMI

^c^Odds ratio for an increase per one unit

^d^Multiple binary regressions considering the terms season and region (longitude)

^e^Multiple binary regressions considering the terms season, region (longitude) and BMI

## Discussion

To our knowledge, these are the first data describing vitamin D status in German breastfeeding women. Three main findings emerged, as follows: (I) 25(OH)D levels were significantly lower in breastfeeding women than in NPNB women; (II) the prevalence of vitamin D deficiency (<25.0 nmol/L) and insufficiency (<50.0 nmol/L) was significantly higher in breastfeeding women compared to NPNB women; and (III) season and longitude influenced the odds of vitamin D deficiency in breastfeeding women.

### Vitamin D status in breastfeeding women compared to NPNB women

A total of 75.8% of breastfeeding women showed insufficient vitamin D status (25(OH)D < 50 nmol/L), while only 5.6% showed optimal vitamin D status (75.0–124.9 nmol/L). It should be noted that the evaluation of vitamin D status depends on the threshold used, and there is only a consensus that 25(OH)D concentrations below 50.0 nmol/L should be avoided [24, 25, 27]. It is estimated that the requirements of 97.5% of the population are adequately covered at 25(OH)D concentrations of at least 50 nmol/L [24]. However the Endocrine Society and other academic experts on vitamin D recommend 25(OH)D concentrations of at least 75.0 nmol/L [5, 25, 27]. Moreover, the vitamin D status may be higher than detected, as we used the DiaSorin Liaison chemiluminescence immunoassay, similar to most previous epidemiological studies [2, 6, 15, 21, 22]. It is known that this method underestimates vitamin D status compared to liquid chromatography tandem mass spectrometry (LC-MS/MS) [34], which has been suggested to be one of the most accurate methods [35].

The vitamin D status was also insufficient (<50 nmol/L) in 58.8% of NPNB women, and this result is comparable with the findings of previous studies of adults in Germany [2, 15, 36]. Food contains only limited amounts of vitamin D [37], which may explain the high proportion of women with vitamin D insufficiency in both groups. On average, German women have a dietary intake of 80–104 IU vitamin D per day [38]. This intake is considerably lower than the recommended daily intake of 800 IU vitamin D per day to ensure 25(OH)D concentrations above 50.0 nmol/L in the case of absent endogenous synthesis [13]. In addition, endogenous vitamin D synthesis via ultraviolet radiation may have been inadequate to fulfill the vitamin D demand for both groups in our study sample. Moreover, none of the women in our study took vitamin D supplements. In the general population of Germany, 32.1% of women use vitamin D supplements; however, the resulting intake is only 156 IU/day on average [39].

Nevertheless, breastfeeding women showed a poorer vitamin D status than NPNB women, with a 4.0 odds of vitamin D deficiency (<25.0 nmol/L) when controlling for potential confounding variables and considering influencing factors.

Recent studies of breastfeeding women in other countries have also shown a high prevalence of inadequate vitamin D status [18, 20, 21]; however, the prevalence was often lower than those rates found in our study [17, 22]. For example, in Sweden, the prevalence of 25(OH)D concentrations < 50 nmol/L in women, who were breastfeeding for 12 months, was 22% in the winter months (November to April) and 15% in the summer months [17]. In a global study of China, the USA and Mexico, 43% of breastfeeding women at 4 weeks postpartum showed vitamin D insufficiency (<50.0 nmol/L) [22]. In contrast to our non-supplementation study sample, in both previous studies, 18% [17] and 22–94% [22] of the women were supplemented with vitamin D. However, in the study sample described by Seth et al., none of the participants supplemented vitamin D, and 93.8% had vitamin D insufficiency [21].

One possible explanation for the lower vitamin D status in breastfeeding women could be the loss of vitamin D via breast milk [32]. In our study, the 25(OH)D level was independent of the duration of breastfeeding, and inconsistent results have been reported in previous studies [17, 32, 40]. The duration of breastfeeding may impact vitamin D status in circumstances of an extended breastfeeding duration (>9 months) [40] and in combination with exclusive breastfeeding [32]. However, we were unable to conclusively and precisely assess the influence of breastfeeding, as the frequency and daily amount of breastfeeding were not evaluated.

Another reason for the lower vitamin D status of breastfeeding women could be that inadequate maternal vitamin D status already existed during pregnancy. A total of 77% of German women showed 25(OH)D concentrations below 50.0 nmol/L after the birth of their child [6]. Jones et al. found a decline of plasma 25(OH)D_3_ from the 30th gestational week in pregnant women to the 12th week postpartum in breastfeeding women, and these results support a lower 25(OH)D_3_ concentration in breastfeeding women than in NPNB women [41].

Moreover, the lower vitamin D status in breastfeeding women may also result from a statistical trend to a higher body weight and BMI among these women. A higher BMI is associated with a higher risk of vitamin D deficiency in the general population [15]. However, the risk of vitamin D deficiency for breastfeeding women was also higher than that for NPNB women in a multivariate adjusted model that included BMI.

### Determinants of vitamin D status in breastfeeding women

In our study, breastfeeding women had similar seasonal variations in the vitamin D status as did NPNB women. However, even between April and September, when exposure may still be sufficient for vitamin D synthesis in northern latitudes [42], this study indicates that 62.3% of breastfeeding women had 25(OH)D concentrations below the sufficient level (<50.0 nmol/L).

Vitamin D synthesis may be dependent upon geography [43]. However, the prevalence of vitamin D deficiency (<25.0 nmol/L) was not associated with latitude in this study, which is in contrast to a previous German study of adults [2] and the fact that the availability of UVB radiation decreases with higher latitude [44]. Interestingly, longitude of residence showed an influence on the vitamin D status. Breastfeeding women who live in lower longitudes of Germany had a higher risk of vitamin D deficiency than breastfeeding women who live in higher longitudes of Germany. This association corresponds to the sunshine duration in Germany, which is associated with longitude instead of latitude. In East Germany (higher longitude) sunshine duration is longer than in West Germany (lower longitude), based on own calculations from the German Meteorological Services data of sunshine duration [45].

In studies of NPNB women, the prevalence of vitamin D deficiency (<25.0 nmol/L) was been found to be significantly lower in those who were non-smokers [46], had lower BMI [46] and had recently traveled to sunny areas [15]. Additionally, lower age [15] and light skin type [47] have been previously found to be associated with higher 25(OH)D concentrations. However, none of these factors were associated with the risk of vitamin D deficiency (<25.0 nmol/L) in breastfeeding women in this study.

### Limitations

Although this is the first data describing vitamin D status in breastfeeding women in Germany, our study sample was not representative and included only a limited number of cases. It is possible that the number of women with potentially confounding characteristics was too low to detect the influence of all associated vitamin D factors. Determinants that may affect vitamin D status, such as dietary vitamin D intake [2], sun exposure and sun protection habits [48], were not evaluated in our study. The absence of this information has two main impacts. First, the influence of longitude on vitamin D status may not have been assessed comprehensively. Second, we cannot exclude that the difference in the vitamin D status between breastfeeding women and NPNP women is influenced by these aspects.

Additionally, our classification schema of skin type (light/dark) may have not been sufficiently precise to determine the influence of skin type on vitamin D status. Moreover, the 25(OH)D concentrations may be higher as detected as the measurement by DiaSorin Liaison chemiluminescence immunoassay underestimate the concentration compared with the analysis by LC-MS/MS [34]. However, the underestimating analysis was applied to both breastfeeding women and NPNB women.

## Conclusion

Our data suggest that an inadequate vitamin D status is prevalent in German breastfeeding women and NPNB women without vitamin D supplementation, even in the summer months. Additionally, breastfeeding women had increased odds of vitamin D deficiency (<25.0 nmol/L) compared with NPNB women. Vitamin D status in breastfeeding women depended on longitude of residence and season at the time of blood collection, with higher 25(OH)D concentrations in summer and autumn than in winter and spring. Because higher maternal vitamin D status may result in a higher vitamin D status of breast milk [8], it may also result in an increased fulfillment of infant vitamin D requirements [14]. Vitamin D containing supplements can be an option to ensure adequate vitamin D concentrations in the absence of personal ultraviolet radiation exposure and low vitamin D intake [13]. However, further studies are necessary to determine the optimal vitamin D status of breastfeeding women and the required vitamin D supplementation doses to reach adequate 25(OH)D levels in this population.

## Abbreviations

25(OH)D: 25-hydroxyvitamin D
BMI: Body mass index
IU: International units
NPNB: Non-pregnant and non-breastfeeding
Ref.: Reference category
VitaMinFemin: Vitamin and mineral status among German women
